# *CHST6* mutation screening and endoplasmatic reticulum stress in macular corneal dystrophy

**DOI:** 10.18632/oncotarget.22028

**Published:** 2017-10-24

**Authors:** Liyuan Wang, Xianling Tang, Xiaolin Lv, Encheng Sun, Donglai Wu, Changlin Wang, Ping Liu

**Affiliations:** ^1^ Eye Hospital, The First Affiliated Hospital of Harbin Medical University, Harbin 150001, China; ^2^ Department of Urology, The First Affiliated Hospital of Harbin Medical University, Harbin 150001, China; ^3^ State Key Laboratory of Veterinary Biotechnology, Harbin Veterinary Research Institute, Chinese Academy of Agricultural Sciences, Harbin 150069, China

**Keywords:** *CHST6*, macular corneal dystrophy, endoplasmic reticulum stress, apoptosis, keratocytes

## Abstract

Macular corneal dystrophy (MCD) is an autosomal recessive disorder mainly caused by gene mutations of carbohydrate sulfotransferase (*CHST6*) leading to bilateral visual impairment. Because the mechanism underlying this degeneration remains poorly understood, we investigated molecular alterations and pathways that may be involved in MCD in this issue. Different mutation sites were screened by direct sequencing of the coding region of *CHST6*. In addition, we described morphological changes in MCD keratocytes by light microscopy and electron microscopy and determined the relationship between the development of this disease and the occurrence of apoptosis through flow cytometry, cell counting kit-8, colony formation assay and other experiments. Western blotting and quantitative real-time polymerase chain reaction were used to determine if endoplasmic reticulum (ER) stress was activated. We found 10 kinds of mutations among these families with 3 novel mutations included. The percentage of apoptotic keratocytes increased in MCD patients; furthermore, the expression of apoptosis related protein B-cell lymphoma-2 (Bcl-2) was down-regulated while Bcl-2 associated X protein was upregulated. Finally, ER stress was activated with the upregulation of glucose-regulated protein 78 and CCAAT-enhancer-binding protein homologous protein. Our clinical and *in vitro* results suggest that the *CHST6* mutation associated with MCD is associated with apoptosis, and ER stress is probably involved in this apoptosis pathway.

## INTRODUCTION

Macular corneal dystrophy (MCD; MIM ^#^217800) is a rare autosomal recessive disorder that usually becomes evident in childhood or adolescence and is clinically characterized by the formation of a diffuse and fine symmetric clouding in the central corneal stroma that extends to the periphery and eventually involves the entire thickness of the cornea, leading to severe bilateral visual disturbance [1, 2]. Currently, corneal transplantation, including penetrating and lamellar keratoplasty, is still the most effective treatment in MCD patients. There are 3 subtypes of MCD (I, IA and II) subdivided by immunoassay and immunohistochemical studies based on the reactivity against an anti-keratan sulfate (KS) antibody (5-D4 anti-KS antibody, AgKS) in the serum and corneal tissues. In type I MCD, AgKS is absent from both serum and corneal tissues, and in type IA MCD, keratocytes manifest AgKS reactivity only. In type II MCD, AgKS reacts positively both to corneal tissues and to serum with normal or subnormal levels [3–5].

Mutations in the carbohydrate sulfotransferase (*CHST6*, OMIM 605294) gene on chromosome 16q22 encoding corneal glucosamine N-acetyl-6-sulfotransferase (C-GlcNAc6ST), an enzyme that transfers sulfate to the unsulfated keratan chains, have been identified as the cause of MCD in humans [6]. With decreased activity of C-GlcNAc6ST, the poorly sulfated or non-sulfated KS is deposited in the intracellular and extracellular matrix of the cornea among MCD patients. Because the sulfation of carbohydrates greatly influences corneal hydration, poorly sulfated and non-sulfated KS loses its hydrophilicity, contributing to corneal opacity and therefore resulting in a loss of visual acuity [7, 8]. Sulfotransferases utilize 3’-phospho-5’-adenylyl sulfate (PAPS) as a sulfonate donor to catalyze the transfer of sulfate to position 6-O of the N-acetyl-glucosamine of keratan in the cornea [6, 9–11]. Mutations in C-GlcNAc6ST domains, such as the 5’PB domain, an essential part of the active site responsible for PAPS binding, seriously effect sulfotransferase activity [12–14].

An increase in apoptotic keratocytes was found in patients’ cornea tissues with macular dystrophy by immunocytochemical analysis of terminal deoxynucleotidyl transferase-mediated dUTP-biotin nick end labeling (TUNEL) assay [15]. Furthermore, apoptotic activity was also detected in epithelial and endothelial cells. In addition, a very low number of p21 positive keratocytes were detected in macular dystrophy corneas but absent in normal cornea tissues [16]. However, the molecular mechanisms and pathways that are triggered result in increased apoptotic keratocyte numbers and remain poorly understood.

According to transmission electron microscopy results, corneas with macular dystrophy revealed a continuous thick basal lamina, and large amounts of characteristic electron-dense and vacuolar deposits were observed in the sub-epithelial stroma [17]. Additionally, the increase in the size of the rough endoplasmic reticulum (ER) was found in keratocytes [18]. The ER is activated by the accumulation of misfolded and unfoldable proteins and the imbalance of Ca^2+^, subsequently termed the unfolded protein response, leading to the increased expression of ER chaperones, such as glucose-regulated protein 78 (GRP78) [19], and downstream molecules, such as CCAAT-enhancer-binding protein homologous protein (CHOP), a key protein for the ER stress-induced apoptosis [20, 21].

Most studies have shown that B-cell lymphoma-2 (Bcl-2) family members control the concentration of ER Ca^2+^ to regulate apoptosis. Bcl-2 was specifically considered an important anti-apoptotic protein regulating the ER Ca^2+^ concentration. In contrast, Bcl-2 associated X protein (Bax) is an important pro-apoptotic protein localized to both mitochondria and the ER, and it promotes Ca^2+^ mobilization from the ER to mitochondria during apoptosis [22–27]. The high expression of CHOP inhibits the expression of Bcl-2, in turn destroying the ratio of Bcl-2/Bax to induce apoptosis [28].

In this study, we identified the underlying genetic defect in 10 Chinese families affected by MCD by undertaking mutation screening of the *CHST6* gene. We identified 3 novel mutations and 7 previously reported mutations consisting of deletions, insertions, missense mutations, and nonsense mutations. We also analyzed the morphology of keratocytes in normal and MCD corneal tissues. In addition, apoptosis and ER stress was further verified in keratocytes with MCD.

## RESULTS

### DNA Analysis

**Figure 1:**
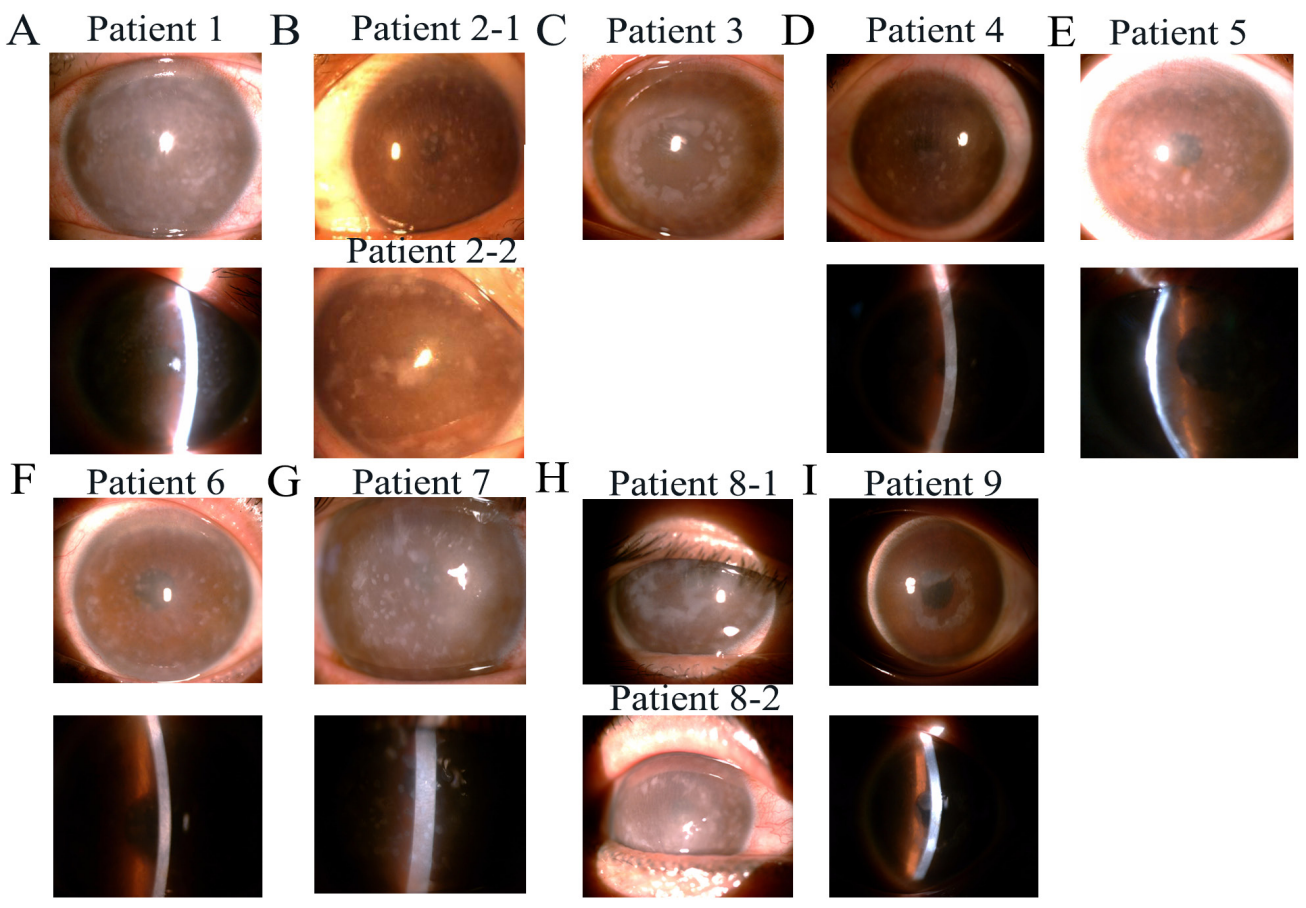
Slit-lamp photographs of patients from nine families with macular corneal dystrophy (MCD) **(A-I)** Showing multiple, irregular gray-white hazes diffused on the central and peripheral cornea. Photograph of family 10 was missing.

Twenty-one affected patients representing 10 distinct genetic families were enrolled in the study. Direct sequencing of the *CHST6* gene from patients with MCD was performed, and 3 novel and 7 previously reported mutations were identified (Table 1). Seven kinds of single base changes and 3 different frame shifts in the coding region of *CHST6* were found in this study; only 2 of them were homozygous. Fifty control chromosomes were analyzed for each alteration by direct sequencing of PCR products, and none of the mutations were found among them. Anterior segment photography showed a number of round gray-white deposits that were diffusely distributed in the corneal stroma in almost all families (Figure 1).

**Table 1:** Ten mutations of CHST6 among ten unrelated Chinese MCD pedigrees

Family	Number of patients	Zygosity	Nucleotide change	Protein change	Mutation type	PolyPhen	SIFT	HGMD NO	Mutation Taster
1	2	Homozygous	c. 382G>A	p. A128T	Missense	0.945/Possibly	0	Novel	DC
2	3	Heterozygous	c. 61-62ins A, 62 T>G	p. L20R fs	Frameshift mutation	MD	MD	Founder	DC
			c. 290-291 ins G	p. S98L fs	Frameshift mutation	MD	MD	Novel	DC
3	3	Heterozygous	c. 418 C>T	p. R140X	Missense	MD	MD	CM050195	DC
			c. 613 C>T	p. R205W	Missense	1.0/Possibly damaging	0	CM104650	DC
4	1	Heterozygous	c. 418 C>T	p. R140X	Missense	MD	MD	CM050195	DC
			c. 1072 T>C	p. Y358H	Missense	1.0/Possibly damaging	0	CM092026	DC
5	1	Homozygous	c. 1072 T>C	p. Y358H	Missense	1.0/Possibly damaging	0	CM092026	DC
6	2	Heterozygous	c. 432C>A	p. S144R	Missense	0.961/Possibly damaging	0.07	CM104653	DC
			c. 463-464 del CG	p. R155Afs	Frameshift mutation	MD	MD	Novel	DC
7	3	Heterozygous	c. 730 G>T	p. E244X	Missense	MD	MD	CM104646	DC
			c. 1072 T>C	p. Y358H	Missense	1.0/Possibly damaging	0	CM092026	DC
8	3	Heterozygous	c. 1072 T>C	p. Y358H	Missense	1.0/Possibly damaging	0	CM092026	DC
			c. 892 C>T	p. Q298X	Missense	MD	MD	CM092027	DC
9	2	Heterozygous	c. 892 C>T	p. Q298X	Missense	MD	MD	CM092027	DC
10	1	Heterozygous	c. 1072 T>C	p. Y358H	Missense	1.0/Possibly damaging	0	CM092026	DC

Predictions and scores estimated with the SIFT, PolyPhen-2 and mutation taster2. MD: must damaging, DC: disease causing.

**Figure 2:**
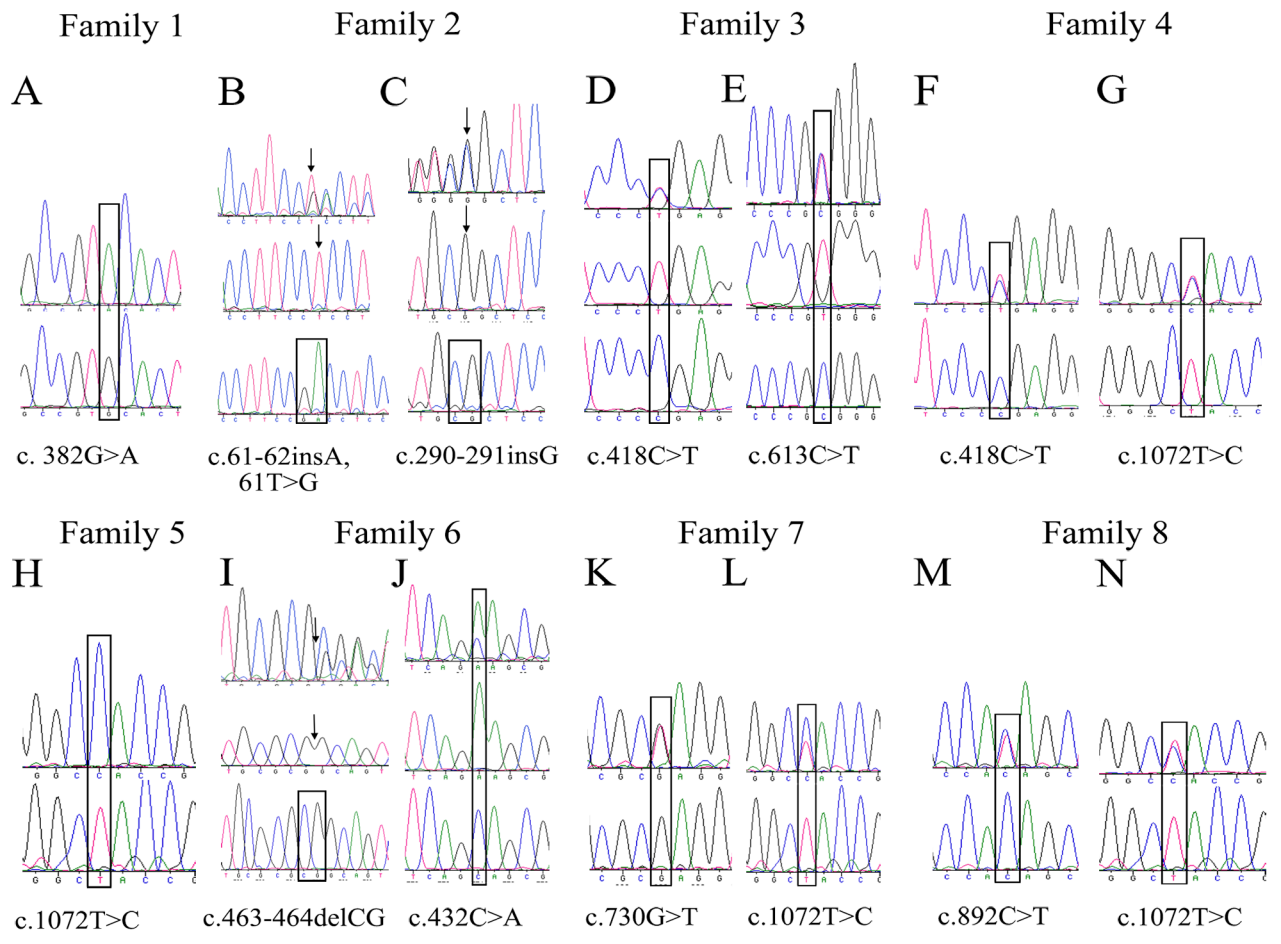
Sequencing chromatograms of variations in *CHST6* identified in this study **(A)** Sequence chromatograms showing one novel homozygous missense mutation in family 1, c.382 G>A. **(B-G, I-N)** sequences of the open reading frame of *CHST6* from heterozygous mutation families were subcloned into p3xFLAG-CMV10 vectors and directly sequenced for heterozygous mutation analysis. **(H)** One founder homozygous missense mutation in family 5, c.1072 T>C.

Patient 1 (Figure 1A) was homozygous for the transition c.382 G>A, which encodes a missense alanine-to-threonine substitution at codon 128 (p.A128T) in family 1 (Figure 2A), and Patient 5 was homozygous for the transition c.1072 T>C, resulting in a tyrosine-to-histidine substitution at codon 358 (p.Y358H) in family 5 (Figure 2H).

Patient 2-1 and patient 2-2 were analyzed in family 2 (Figure 1B). Two compound frame shift changes were identified by sequencing. The results showed an insertion of a single base pair between nucleotides 290 and 291, resulting in a frame shift after codon S98 (p.S98Lfs) (Figure 2C). Another frameshift mutation was found after codon 20, depending on a base pair insertion of adenine (insA) after the transversion of thymine to guanine at nucleotide position 62 (c.62 T>G) (Figure 2B).

Both the brother and sister of family 6 inherited MCD and showed the same symptoms. The brother underwent penetrating keratoplasty because of numerous spot deposits diffused on all layers of the cornea; however, the sister only went through lamellar keratoplasty without involving the endothelium layer (Figure 1F). One heterozygous mutation with both a frame shift change and a single base nucleotide change was found in family 6 (denoted the c.463-464 delCG novel variant). These changes result in a frame shift after codon 155 (p.R155Afs) (Figure 2I) and a single base nucleotide variant c.432 C>A (p.S144R) (Figure 2J).

Furthermore, heterozygosity with compound single base nucleotide changes was detected in 6 other families. In family 3, irregular large spots were seen in the proband's cornea (Figure 1C), and a heterozygous change, c.418 C>T (Figure 2D) and c.613 C>T (Figure 2E), was identified, predicting amino acid changes of an arginine to a stop codon (p.R140X) and arginine to a tryptophan (p.R205W). Heterozygous mutants including c.730 G>T (Figure 2K) and c.1072 T>C (Figure 2L), which predicted amino acid changes of arginine to a stop codon (p.E244X) and tyrosine to histidine (p.Y358H), respectively, were identified in family 7 (Figure 1G). In addition, a heterozygous change, c.418 C>T (Figure 2F) and c.1072 T>C (Figure 2G), resulting in glutamine and tyrosine changing to a stop codon (p.R140X) and histidine (p.Y358H), respectively, was found in family 4 (Figure 1D). Additionally, a heterozygous change, c.892 C>T (Figure 2M) and c.1072 T>C (Figure 2N), leading to amino acid changes of glutamine to a stop codon (p.Q298X) and tyrosine to histidine (p.Y358H), was detected in family 8 (Figure 1H).

In families 9 and 10, only one heterozygous pathogenic change was observed in the coding *CHST6* sequence. We found c.1072 T>C, which changes a tyrosine to a histidine (p.Y358H) in patient 9 with white spots developing in sheets in the cornea (Figure 1I). Additionally, c.892 C>T was found in family 10, changing a glutamine to a stop codon substitution (p.Q298X).

When the pathogenic effect of the novel missense variation was evaluated with SIFT and PolyPhen-2 in silico analysis software, the results were “probably damaging” and “affect protein function,” respectively (Table 1). The 3 novel mutations including missense and frameshift variations were also tested with MutationTaster, which predicted “disease causing”. Additionally, all newly detected variants in the *CHST6* gene were not found in 50 control chromosomes, 1,000 Genomes (1000G), Exome Aggregation Consortium (ExAC), and the Human Gene Mutation Database (HGMD). Furthermore, amino acid sequence analyses between humans and other mammals revealed that the amino acids substituted in the missense variation detected in the patients were highly conserved residues.

### Histologic and ultrastructural changes in corneas with MCD

**Figure 3:**
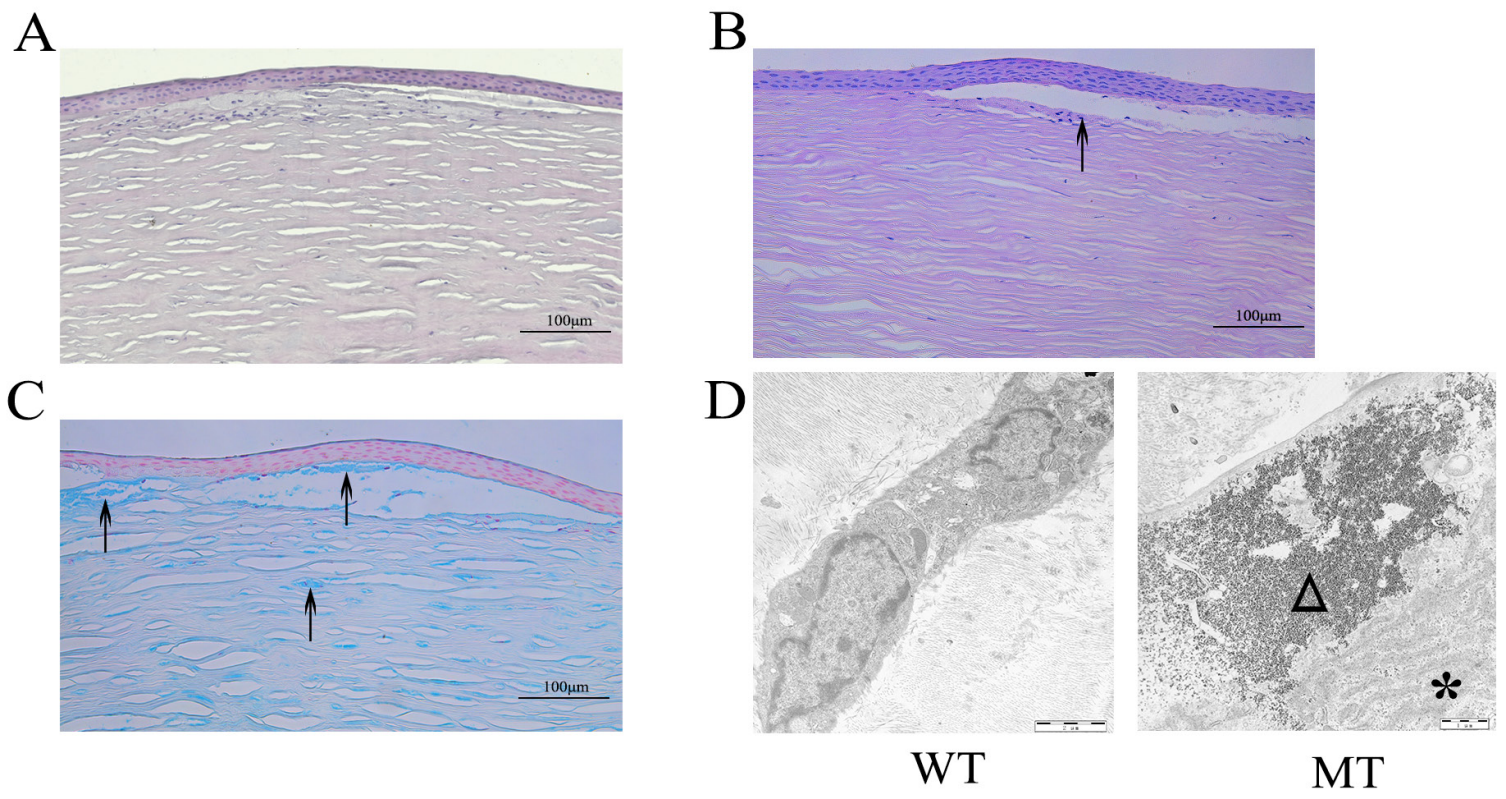
Pathological changes of MCD tissues observed by histologic and ultrastructural analysis **(A)** Subepithelial collagen fibers showed irregularity with hyalin deposited, stained with hematoxylin and eosin. **(B-C)** PAS and Alcian blue stain showed abnormal accumulations in the subepithelial and upper layer of the stroma. **(D)** Ultrastructural analysis showed that a large number of electron dense deposits are deposited in the corneal stroma with activated rough ER. Arrows show the polysaccharide sulfate deposits. The asterisked triangle represents the rough ER. Triangles indicate the electron dense deposits.

By light microscopy, there were great changes in the morphology and structure of the epithelium and stroma between the MCD and normal corneas. The epithelium of corneas with deposit areas was thin and irregularly arranged, decreasing from 5-6 to 3-4 cell layers. On the other hand, the disordered arrangement of collagen fiber and increased density of keratocytes were seen in the subepithelial and upper layer of the stroma according to the hematoxylin and eosin staining results (Figure 3A). In five corneal buttons with macular dystrophy, PAS and Alcian blue staining revealed typical polysaccharide sulfate deposits intracellular and extracellular in the subepithelial layer and stroma (Figure 3B, 3C). By transmission electron microscopy, in the stroma of MCD corneas, a large number of electron dense granules were deposited in the cytoplasm of the keratocytes with a large number of the rough ER surrounded (Figure 3D), which was not detected in the normal stroma.

### Characterization of MCD keratocytes

**Figure 4:**
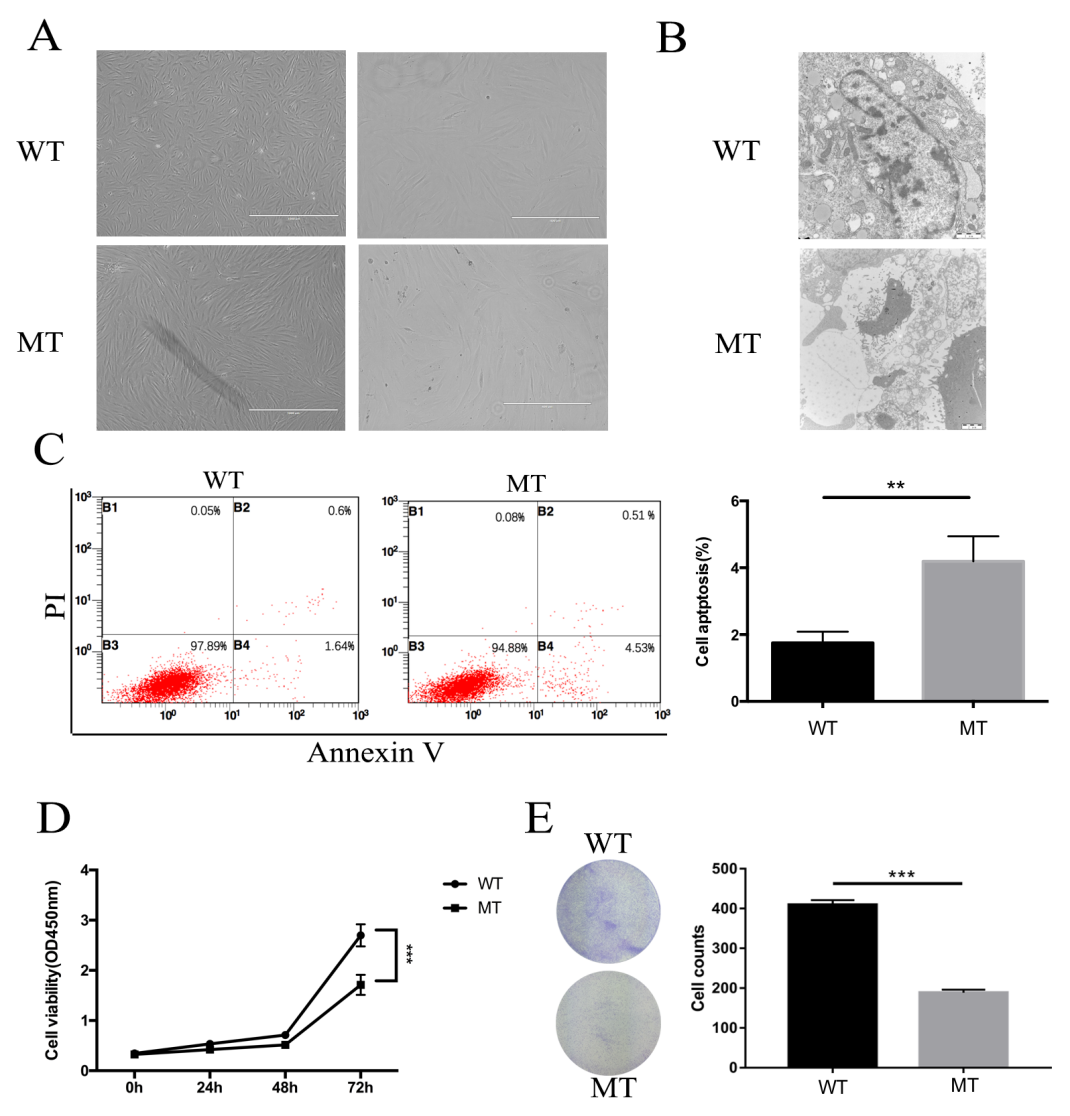
The changes in proliferation and apoptosis in MCD keratocytes **(A)** Keratocytes from patients were larger, longer and included more sick cells. **(B)** Characterization of apoptosis of MCD keratocytes analyzed by transmission electron microscope examination. Concentration of encapsulated cells, chromosome aggregation and apoptosis body formation are shown. **(C)** Flow cytometry results show that the percentage of apoptotic cells increased in the MT group. **(D-E)** Cell proliferation and clonogenic assays illustrated that keratocytes of MT had better cell viability and a stronger ability to form clones than WT. Statistical analysis between the two groups were analyzed by t-test, in which a p-value of less than 0.05 was considered statistically significant (^**^ p<0.01, ^***^p<0.001).

The morphological changes between WT and MCD keratocytes were analyzed by phase contrast microscopy. Through comparative analysis, MCD keratocytes were larger in size, and showed a senescence-like morphology, which was not seen in WT keratocytes (Figure 4A). These morphological abnormalities often suggested that certain pathological changes occurred on MCD keratocytes due to the mutations. Ultrastructural analyses using electron microscopy were used to reveal characteristics of the apoptotic MCD keratocytes in which cells became crinkled, chromatin aggregated and apoptotic bodies formed (Figure 4B).

### Keratocyte proliferation and apoptosis in MCD

The primary cultured WT and MT keratocytes subcultured for 6-10 generations were used for flow cytometry. The MT group exhibited an increase in apoptotic cells (Figure 4C). Furthermore, cell proliferation assays showed that the growth in MCD keratocytes was inhibited significantly compared with the normal keratocytes (Figure 4D). We also did a clonogenic assay and found that clone numbers in the MCD keratocytes were reduced significantly (Figure 4E).

### ER stress in response to mutation of *CHST6* in MCD keratocytes

**Figure 5:**
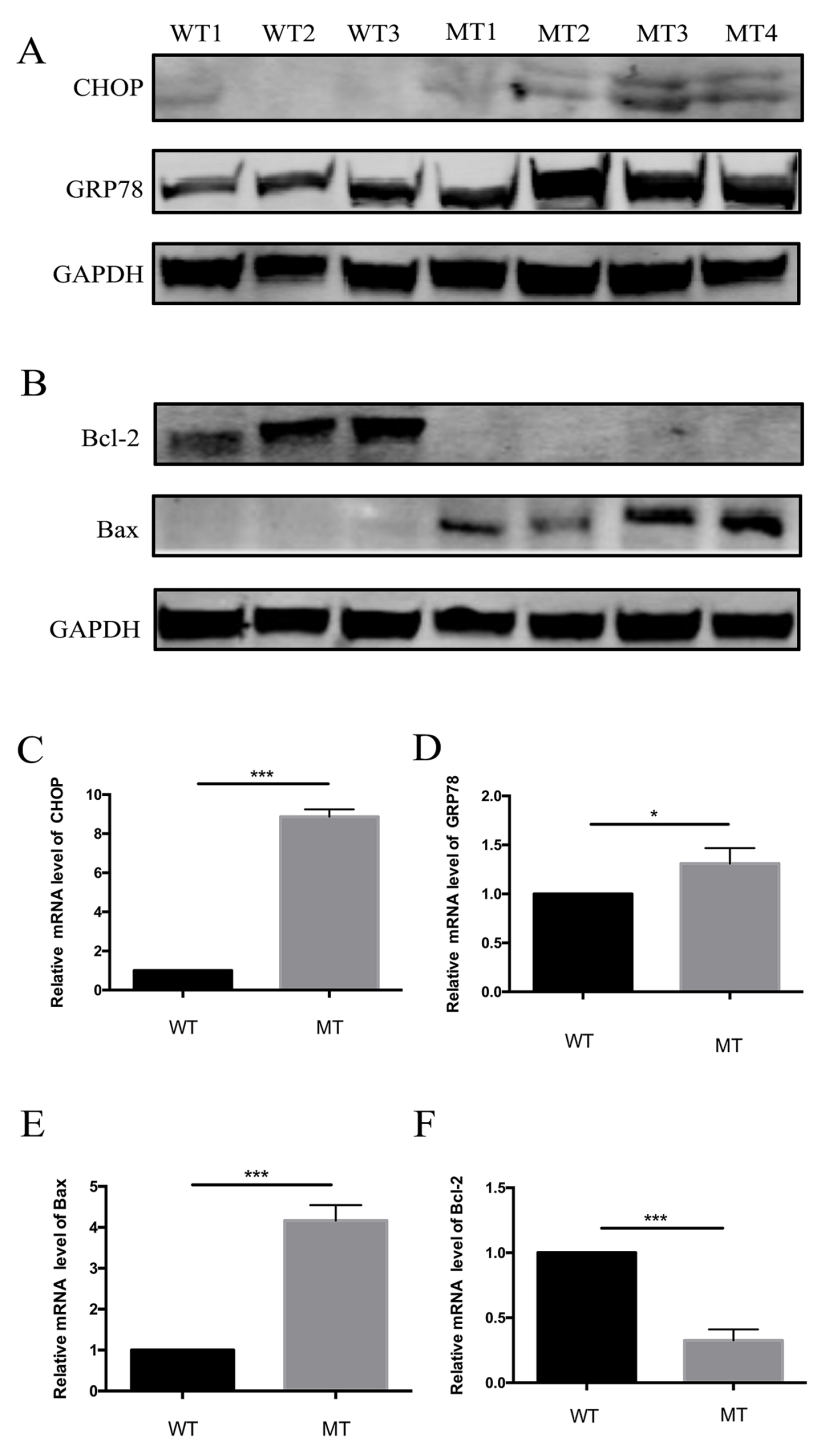
Expression of regulation proteins of apoptosis and ER stress pathways in WT and MT keratocytes **(A)** Expression of CHOP, an ER stress-associated transcription factor, and GRP78, an ER chaperone. **(B)** Expression of Bcl-2 family proteins in WT and MT keratocytes. Bcl-2 was downregulated, but Bax was upregulated. **(C-F)** Relative mRNA levels of CHOP, GRP78, Bcl-2 and Bax. Only Bcl-2 was decreased (* p<0.05, ^***^ p<0.001).

We proposed that apoptosis occurred by the activation of ER stress in MCD keratocytes. To assess the effects of ER stress in MCD keratocytes, we detected the expression of GRP78 by western blotting and qPCR. As depicted, GRP78 was significantly upregulated in MCD keratocytes, compared to the control (Figure 5A, 5D). We then verified the expression of CHOP, a key protein for ER stress-induced apoptosis, through western blotting and real-time PCR. The results clearly showed that CHOP was upregulated in MCD keratocytes according to statistical analysis (Figure 5A, 5C). Finally, changes in Bcl-2 and Bax were compared to verify if apoptosis occurred in keratocytes with *CHST6* mutations. It appeared that Bcl-2 levels were much lower in MCD cells than in normal cells (Figure 5B, 5F). In contrast to the attenuated expression of Bcl-2, that of Bax, a pro-apoptotic protein, was enhanced in MCD keratocytes (Figure 5B, 5E).

## DISCUSSION

In this study, we report the mutation screening of the *CHST6* gene in 10 Chinese families with MCD. Ten independent mutations, seven missense, two insertions and one deletion were identified. Of these, three were novel, whereas the others were founded previously in MCD patients [14]. Furthermore, we preliminary investigated the role of ER stress and apoptosis in MCD keratocytes.

Genetic mutations at different sites most likely affect the function of the gene, especially at positions in the protein that are highly conserved across carbohydrate sulfotransferases and/or within important domains that are essential for maintaining the structure and function of the protein. For example, the substitution of p.R205W, located within the 5’PB domain, seriously reduces the ability to combine with PAPS. This affects the sulfide transfer function of sulfotransferases [10, 13]. In addition, 2 missense mutations (p.S144R and p.Y358H) affected enzyme activity and involved the substitution of an uncharged polar residue for a basic residue at a non-conservative or conservative site. Although the previously unreported p.A128T substitution belonged to a chemically similar amino acid, its position was highly conserved in the GlcNAc6ST gene family, such that a mutation at that position makes C-GlcNAc6ST inactive. On the other hand, we have discovered one deletion, two insertions and three nonsense mutations of stop codons. These mutations were all predicted to terminate the translation in advance with premature stop codons. Abnormal mRNAs of GlcNAc6ST, including premature termination codons, are eliminated by the physiological process of nonsense-mediated decay [29].

ER stress is a universal physiopathological response to a variety of stimuli that may play a protective role or may induce cell apoptosis independently. Our results showed that the ER stress marker protein GRP78 is upregulated in MCD keratocytes, which shows that the ER stress response is active. On the other hand, the upregulation of GRP78 is a key step leading to subsequent cellular events including cell death [30, 31]. We confirmed that the expression of CHOP was enhanced by western blotting and real-time PCR (Figure 5). Activated CHOP could influence gene expression related to apoptosis, including decreased Bcl-2 expression [32], which was consistent with our experimental results.

As described by Szentmáry there is a statistically significant increase in apoptotic keratocyte numbers in corneal tissues with macular dystrophy [16]; keratocytes from MCD patients also have this feature verified in this article. In our results, the expression of Bcl-2 was significantly reduced, whereas Bax was increased. The ratio of Bcl-2/Bax decreased significantly in MCD keratocytes compared with normal cells, thus inducing apoptosis.

In summary, based on our findings, we not only identified 10 mutations including 3 novel mutations but were also able to prove that apoptotic cell death was increased in keratocytes from MCD patients. A striking finding of this study is that mutations in *CHST6* may trigger ER stress with considerable GRP78/CHOP upregulation and cell apoptosis, however, the exact molecular mechanisms need to be validated by more experiments. There are no effective treatments for this hereditary MCD except for corneal transplantation. By studying the pathological process of MCD and interfering with the signaling pathway, MCD could be delayed or even blocked.

## MATERIALS AND METHODS

### Patients and control subjects

**Figure 6:**
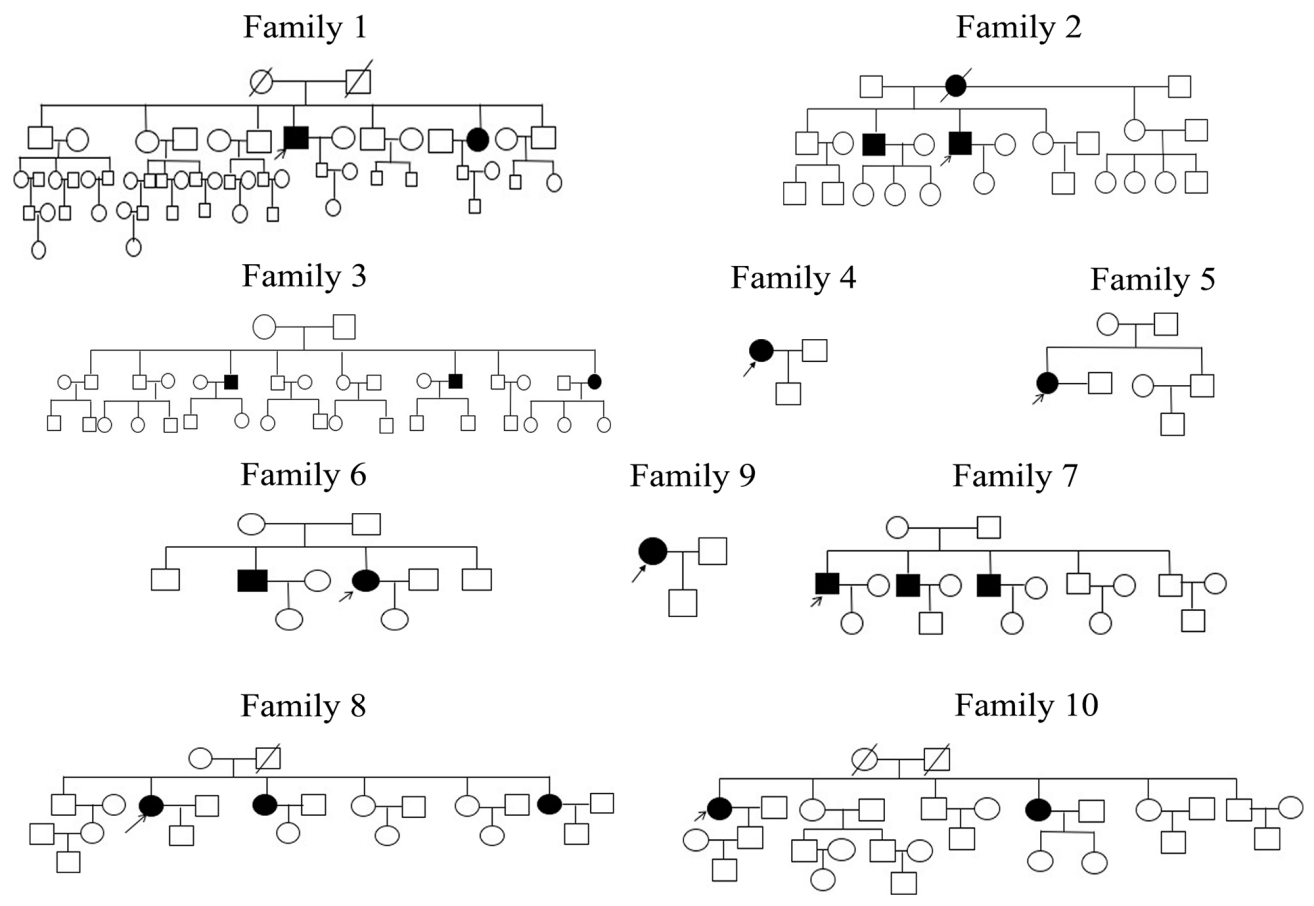
Ten MCD families who participated in the study Open and closed symbols indicate unaffected and affected individuals, respectively. Deceased family members are denoted by diagonal slashes, the arrow marks the proband in these families.

Twenty-one patients from 10 unrelated families (Figure 6) received clinical diagnoses of MCD by three doctors using slit-lamp biomicroscopy in the First Affiliated Hospital of Harbin Medical University; corneal optical coherence tomography was also analyzed in this study. Control subjects were selected from 50 individuals with no visual impairment. The diagnosis in five patient families was MCD type I. Informed consent was obtained from all patients and their family members in agreement with the Declaration of Helsinki for research involving human subjects. Approvals for genetic testing were obtained from the First Affiliated Hospital of Harbin Medical University.

### *CHST6* analysis

Genomic DNA was isolated from peripheral blood leukocytes by standard techniques according to the manufacturer's instructions (Qiagen, USA). Three pairs of primers designed by Akama et al. were used to amplify the region of the open reading frame (ORF) of *CHST6* [6]. Each PCR reaction was performed as a 50 μl reaction mixture consisting of genomic DNA (100 ng) and 25 μl of PrimeSTAR HS Premix DNA polymerase (Takara, Japan) containing PrimeSTAR HS DNA Polymerase, dNTP Mixture and PrimeSTAR HS Buffer. Amplification reactions were performed under the following conditions: 5 min of denaturation at 98°C followed by 35 cycles of denaturation at 98°C for 10 seconds, annealing for 15 seconds at 55°C (for the middle coding region) or at 57°C (for the 5’ and 3’ coding regions), extension at 72°C for 45 seconds, and a further extension step at 72°C for 7 min. The PCR products were separated by agarose gel electrophoresis and purified with a kit (Gel Extraction Kit; OMEGA, USA). Bidirectional sequencing was performed by using a DNA sequencer (model 3730; Applied Biosystems, CA, USA). The ORFs of *CHST6* of 10 families were cloned into p3xFLAG-CMV10 vector by PCR product using the primers, F: 5’AGTCGAATTC AATGTGGCTGCCGCGCGTC3’ and R: 5’ ACTGTCTAGACTAATTTCGGGGGTGCGAGGC3’. The resulting plasmid was named p3xFLAG-*CHST6*. All PCR products and plasmids were sequenced for fidelity.

The pathogenicity of the novel missense variation was evaluated with the SIFT and PolyPhen-2 software programs. Additionally, conservation of the involved amino acids among several kinds of mammals was evaluated using MutationTaster.

### Histologic and transmission electron microscopy of corneal tissues and keratocytes

Corneal buttons obtained from MCD patients who underwent keratoplasty were processed for histologic and transmission electron microscopy (EM) studies. Histologic analysis was performed on 4 μm thick sections of paraffin-embedded cornea buttons from 5 unrelated MCD patients who underwent keratoplasty. Hematoxylin and eosin (HE), periodic acid-Schiff (PAS), and Alcian blue were used to stain the corneal tissues for light microscopy analysis. In addition, keratocytes and corneal tissues were prepared for thin sectioning. Briefly, the specimen was fixed with 2.5% (vol./vol.) glutaraldehyde in 0.1 M for 2 h, rinsed with 3 changes of phosphate-buffered saline (PBS; Gibco BRL, Grand Island, NY), and postfixed with 1% (vol./vol.) OsO_4_ in PBS for 2 h. After being washed, the specimen was dehydrated in a graded series of ethanol and embedded in epoxy (low-viscosity agar) resin, following the standard protocol. Ultrathin sections were collected on carbon-coated 100 mesh copper grids and stained with 1% uranyl acetate and 1% lead citrate. EM grids were screened at 80 kV in a Hitach-H7650 transmission electron microscope.

### Culture of primary keratocytes

Primary keratocytes were acquired from wild type (WT) donors from the Heilongjiang Province Eye Bank and from MCD patients, mutation type group (MT), after penetrating or lamellar keratoplasty. Corneas were washed 3 times, 15 min per time, with 1 × PBS containing 1000 units/ml penicillin and 1.0 mg/ml streptomycin sulfate (Gibco BRL, Grand Island, NY). The corneal epithelium, endothelium, sclera and conjunctiva were removed completely. A quarter of the corneal tissues were cultured on 30-mm culture dishes with 200 μl of 10% fetal bovine serum F12/DMEM containing 1000 units/ml penicillin and 1.0 mg/ml streptomycin sulfate overnight at 37°C in 5% CO_2_, followed by incubation in 500 μl of medium. The primary keratocytes were subcultured to 90% confluency in media containing 0.25% trypsin and 5.0 mmol/L EDTA.

### Flow cytometry and apoptotic analysis

Annexin V/dead cell apoptosis analysis was performed according to the manufacturer's protocol (Alexa Fluor® 488 Annexin V/Dead Cell Apoptosis Kit, Invitrogen, USA). Approximately 1×10^5^ keratocytes per well were plated onto 9.6 cm^2^ cell culture dishes. Then, 18-24 h later, 1×10^6^ keratocytes were harvested after washing in cold PBS and re-centrifuged with 100 μl 1× Annexin-binding buffer. Five microliters of Alexa Fluor® 488 Annexin V and 1 μl of 100 μg/mL propidium iodide (PI) working solution were then added to each 100 μl suspension of keratocytes and incubated at room temperature for 15 min. After incubation, 400 μl of 1×Annexin-binding buffer was added, mixed gently and stored on ice. We analyzed the stained cells by flow cytometry (Cytomics TM FC 500, Beckman Coulter, USA).

### Cell proliferation assay

Cells were detached by treatment with 0.25% trypsin-EDTA (Invitrogen, Carlsbad, CA, USA) and seeded into 96-well plates at a density of 1×10^3^ per well in 100 μl of media. For the CCK-8 assay, a Cell Counting Kit-8 (Beyotime, Shanghai, China) was used following the manufacturer's instructions. Briefly, 2 h before each of the desired time points (24 h, 48 h and 72 h), the CCK-8 reagent was added to each well at a dilution of 1:10, and cells were incubated at 37°C for 2 h. The absorbance was detected at 450 nm using a microplate reader (BioTek, VT, United States). All of the experiments were repeated three times.

### Clonogenic assay

For the clonogenic assay, 5000 cells per well were seeded in 6-well plates. Visible colonies were observed using the naked eye 14 days later, fixed with 4% formaldehyde, and stained with 0.1% crystal violet. Colonies with a diameter greater than 1 mm were counted.

### Real-time quantitative reverse transcription-PCR

A two-step reaction process was used for quantification: reverse transcription (RT) and PCR. The ReverTra Ace qPCR RT Kit (TOYOBO, Japan) was used for RT according to the manufacturer's protocols. The RT reaction consisted of 1 μg of RNA, 2 μl of 5 × RT Buffer, 0.5 μl of primer mix, 0.5 μl of RT Enzyme Mix and 6 μl of nuclease-free water in a total volume of 10 μl. Reactions were performed in an Eppendorf PCR System (Eppendorf, Germany) for 15 min at 37°C, followed by heat inactivation for 5 min at 95°C. Then, the 10 μl RT reaction mix was held at -20°C.

A 480 II Real-time PCR Instrument (Roche, Basel, Switzerland) was used with a 20 μl PCR reaction mixture that included 2 μl of cDNA, 10 μl of 2 × LightCycler® 480 SYBR Green Realtime PCR Master Mix (TOYOBO, Japan), 0.8 μl of forward primer, 0.8 μl of reverse primer and 6.4 μl of nuclease-free water. Reactions were incubated in a 96-well optical plate (Roche) at 95°C for 30 seconds; followed by 40 cycles of 95°C for 5 seconds, 55°C for 10 seconds, and 72°C for 15 seconds. Each sample was run in triplicate for analysis. At the end of the PCR cycles, melting curve analysis was performed to validate the specific generation of the expected PCR product. All experiments were done in triplicate. The expression levels of RNAs were normalized to glyceraldehyde-3-phosphate dehydrogenase (GAPDH) and were calculated using the 2^-ΔΔCt^ method [22]. The primer sequences were designed in the laboratory and synthesized by Sangon Biotech (Sangon, Shanghai, PRC).

### Western blotting

Cells were collected and lysed in RIPA buffer supplemented with 1% PMSF. Protein was separated on a 12% sodium dodecyl sulfate-polyacrylamide gel electrophoresis (SDS-PAGE) gel and subsequently transferred to polyvinylidene difluoride (PVDF) membranes (Millipore Corp, Atlanta, GA, US). After blocking with 5% non-fat dry milk, the PVDF membranes were incubated with the following primary antibodies overnight at 4°C: rabbit polyclonal anti-GRP78 (Abcam, MA, USA), anti-CHOP (Santa Cruz, CA, USA), anti-GAPDH (Proteintech, IL, USA), anti-Bcl-2 (Santa Cruz, CA, USA), anti-Bax (Santa Cruz, CA, USA). Membranes were incubated with an IRDyeTM 700DX -conjugated secondary antibody, Donkey Anti-Rabbit IgG (H+L) (LI-COR, NE, USA), at room temperature for 1 h. The signal was detected and analyzed by a LI-COR Odyssey infrared imaging system.

### Statistical analysis

All statistical analyses were performed using GraphPad software (GraphPad Prism 6, GraphPad Software, La Jolla, CA, USA). The differences between groups were compared by using Student's t-test for all *in vitro* studies. A p<0.05 was considered significant (* p<0.05, ^**^ p<0.01, ^***^ p<0.001).

## Abbreviations

MCD: macular corneal dystrophy
WT: wild type
MT: mutation type
*CHST6*: carbohydrate sulfotransferase
C-GlcNAc6ST: corneal glucosamine N-acetyl-6-sulfotransferase
KS: keratan sulfate
Bcl-2: B-cell lymphoma-2
Bax: Bcl-2 Associated X Protein
ER: endoplasmic reticulum
GRP78: glucose-regulated protein 78
CHOP: (CCAAT-enhancer-binding protein homologous protein)
GAPDH: glyceraldeyde-3-phosphate dehydrogease
ORF: open reading frame
PAPS: 3’-phospho-5’-adenylyl sulfate
PMSF: phenylmethylsulfonyl uoride
CCK-8: Cell Counting Kit-8
qPCR: quantitative real-time Polymerase Chain Reaction
TUNEL: terminal deoxynucleotidyl transferase-mediated dUTP-biotin nick end labeling assay
HE: Hematoxylin and eosin
PAS: periodic acid-Schiff
PBS: phosphate-buffered saline
EM: electron microscopy
EDTA: Ethylene Diamine Tetraacetie Acid
DMEM/F12: Dulbecco's Modified Eagle Media: Nutrient Mixture F-12
PI: propidium iodide
PVDF: polyvinylidene difluoride
SDS-PAGE: sodium dodecyl sulfate-polyacrylamide gel electrophoresis
